# *Bartonella quintana* Endocarditis and Pauci-Immune Glomerulonephritis in Patient without Known Risk Factors, USA, 2024

**DOI:** 10.3201/eid3104.241812

**Published:** 2025-04

**Authors:** Weibo Yu, Christopher N. Tymchuk, Ran Zhuo, Daniel P. Stefanko, Cody Forsyth, Colette J. Matysiak Match, Sukantha Chandrasekaran, Gregory A. Fishbein, Shangxin Yang

**Affiliations:** UCLA David Geffen School of Medicine, University of California Los Angeles, Los Angeles, California, USA

**Keywords:** *Bartonella quintana*, bacteria, endocarditis, glomerulonephritis, California, United States

## Abstract

We report an unexpected case of *Bartonella quintana* endocarditis and pauci-immune glomerulonephritis in a patient without known risk factors in Los Angeles, California, USA, highlighting that infection can occur in the general population without a history of homelessness. The diagnosis was challenging and made definitively through extensive diagnostic tests and multidisciplinary investigation.

*Bartonella* spp. are intracellular, gram-negative, aerobic coccobacilli bacterium that are fastidious and require special conditions for growth; they are not identified by routine blood culture. *B. quintana*, the cause of trench fever, and *B*. *henselae*, the cause of cat-scratch disease, are the 2 most common species causing *Bartonella* endocarditis (*1*). Patients with *Bartonella* endocarditis often seek care for nonspecific symptoms and a chronic clinical course. Because of cross-reactivity in serologic tests and frequent negative cultures, initial diagnosis and pathogen identification for *Bartonella* endocarditis can be challenging (*2*). In addition, the morphologic diversity of *Bartonella* in histopathologic exams may mimic other bacteria, further complicating identification (*3*).

*B*. *quintana* is transmitted by the human body louse. It was first described during World War I but was found to be prevalent in the urban homeless population in industrialized countries in the early 1990s, likely because of advances in the application of molecular diagnostic techniques (*4*–*6*). California, USA, has relatively high rates of *B*. *quintana* infections, mainly associated with persons experiencing homelessness because of crowded living conditions, limited access to healthcare, and poor hygiene (*6*–*8*).

In this article, we report a case of culture-negative *B*. *quintana* endocarditis, in which the Gram stain initially revealed gram-positive cocci on the mitral valve. Pauci-immune glomerulonephritis developed first in the patient, which was likely also caused by *B*. *quintana* infection. The final identification was confirmed through a combination of intraoperative findings, pathologic examination, serologic testing, and molecular testing, including next-generation sequencing (NGS).

## The Study

A 48-year-old man with a 1-year history of mitral regurgitation (MR), mitral myxomatous disease, pauci-immune glomerulonephritis, IgA nephropathy on daily cyclophosphamide, and hypertension sought care for a mitral valve repair. Anti-neutrophil cytoplasmic antibody (ANCA) tests showed highly elevated c-ANCA at 1:320 (PR3 positive and MPO negative) and negative p-ANCA. The patient reported no abnormalities and had no prior symptoms of endocarditis. He underwent a dental procedure (2 cavity fillings and 1 extraction) before admission and received clindamycin before the procedure. The patient had no history of homelessness or of intravenous drug or other recreational substance use and had no exposure to large animals or pets, including cats. The patient was born in Canada and immigrated to the United States 5 years earlier. He lives in an apartment and works in information technology. He does not have any exposure to homeless shelters and does not have any history of louse infestations.

**Figure 1:**
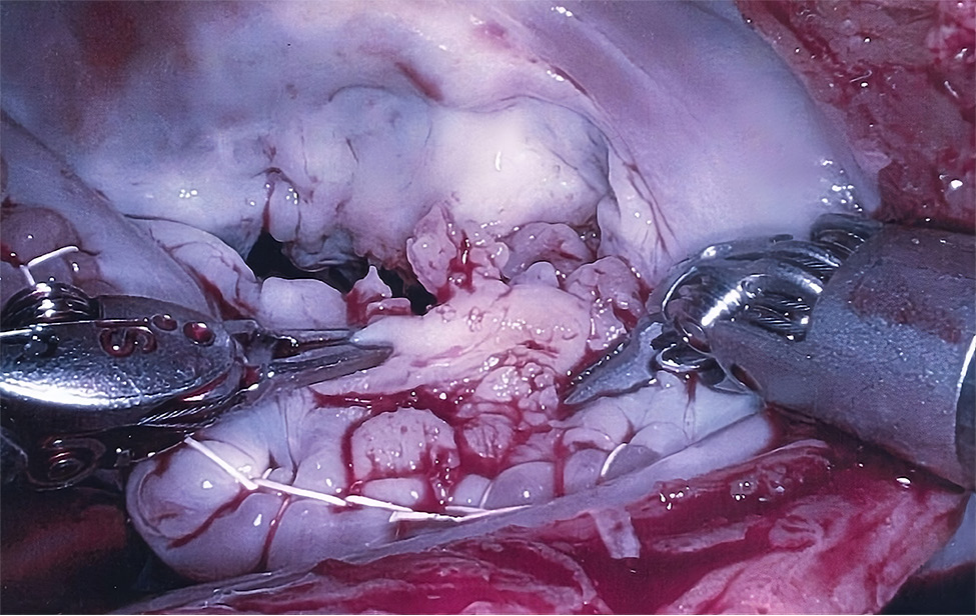
Intraoperative image showing verrucous excrescences on the mitral valve from a patient with *Bartonella quintana* endocarditis and pauci-immune glomerulonephritis, California, USA.

**Figure 2:**
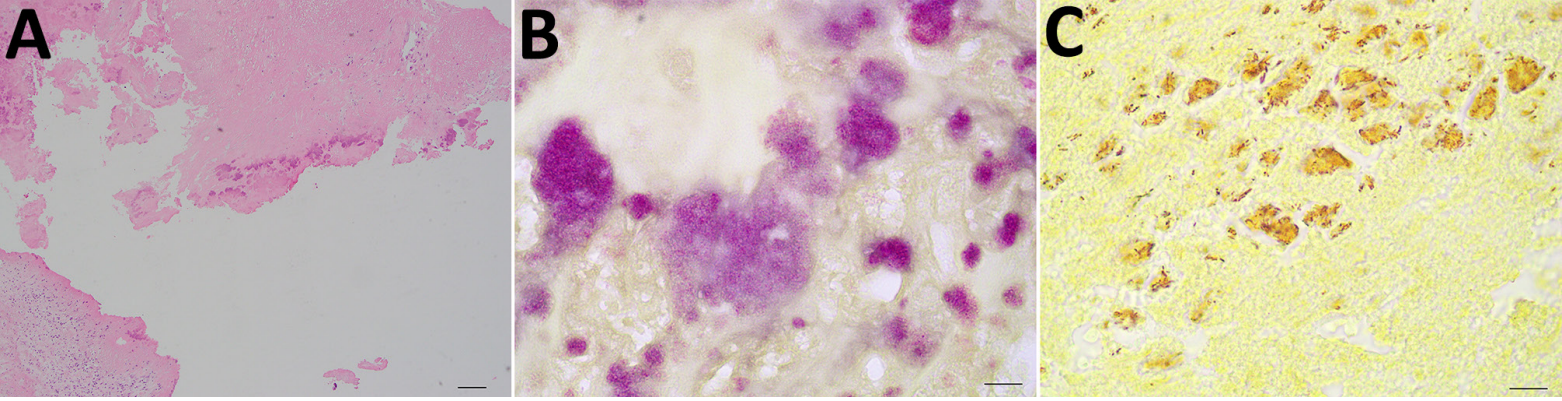
Histopathology images from a patient with *Bartonella quintana* endocarditis and pauci-immune glomerulonephritis, California, USA. A) Hematoxylin and eosin stain revealing infiltration of neutrophils and lymphocytes as well as precipitation of fibrin. B) Gram stain showing numerous degenerated bacteria clumped together, mimicking gram-positive cocci. C) Warthin-Starry stain showing numerous rod-shaped bacteria.

Because of severe MR, the patient underwent mitral valvuloplasty. During the procedure, unexpected verrucous excrescences on the mitral valve were noted, and debridement of the mitral valve lesions was conducted (Figure 1). The initial histopathologic examination of the valve tissue stained with hematoxylin and eosin revealed infiltration of neutrophils and lymphocytes, as well as fibrin precipitation, which were consistent with active infective endocarditis (Figure 2, panel A). Grocott methenamine silver and acid-fast bacteria stains were negative for fungal and mycobacterial organisms. The Gram stain suggested possible gram-positive cocci (Figure 2, panel B). Because of the positive pathological findings, we started the patient on empiric treatment with ceftriaxone and vancomycin. Blood and valve tissue cultures returned negative results 6 days after the procedure, but a plasma Karius test was positive for *B*. *quintana* (3,611 molecules/mL). We sent the indirect fluorescent antibody tests to ARUP Laboratories (https://www.aruplab.com), which confirmed the positive *B*. *quintana* IgG in the blood with a titer of >1:1,024. As expected, the *B*. *henselae* IgG test also showed a titer of >1:1,024 due to cross-reactivity. Because of those results, we discontinued vancomycin and initiated doxycycline (100 mg orally 2×/d) and a 4-week course of rifampin (300 mg orally 2×/d).

To confirm the Karius test and serologic results, we conducted a Warthin-Starry stain and submitted the specimen for 16S rRNA and internal transcribed spacer amplicon-based targeted NGS testing (*9*,*10*) and to the Centers for Disease Control and Prevention for additional molecular testing. The Warthin-Starry stain highlighted focal clusters of coccobacilli (Figure 2, panel C). Both the targeted NGS and additional molecular testing confirmed the presence of *B*. *quintana*. The consensus 16S rRNA sequence generated by the targeted NGS matched 100% to *B*. *quintana* by BLAST (https://blast.ncbi.nlm.nih.gov).

Four months after the procedure, the patient completed a 12-week course of doxycycline. Positron emission tomography–computed tomography results demonstrated no evidence of mitral valve infection. A repeat Karius test was negative for *Bartonella* spp. However, a *B*. *quintana* IgG titer remained high at >1:1,024. The patient’s c-ANCA level dropped to 1:20, accompanied by a nearly full clinical resolution of the pauci-immune glomerulonephritis. The patient reported feeling overall better, continued taking doxycycline, and remained under clinical follow-up monitoring.

## Conclusions

In this case, endocarditis was not diagnosed until after cardiac surgery and valve replacement. The source of the *B*. *quintana* infection was unclear because cases were previously reported only from persons experiencing homelessness in California (*6*). The patient, who was receiving immunosuppressive therapy, might have had *B*. *quintana* endocarditis after a chronic infection that initially did not manifest with noticeable symptoms. The final diagnosis was established through intraoperative findings, pathology examination, serologic testing, and multiple NGS-based tests.

Of note, the initial Gram stain showed gram-positive cocci on the mitral valve. Because *B*. *quintana* is gram-negative, this finding raised questions of possible variability in Gram staining or a mixed infection. The patient had a recent dental procedure with fillings and an extraction, which could have seeded the bloodstream with a *Streptococcus* species, potentially affecting the valve. However, other bacterial species were not detected by either Centers for Disease Control and Prevention testing or our targeted NGS tests. Of note, Gram stain is not a sensitive or reliable test for *Bartonella* spp., and therefore, histopathologic morphology of *Bartonella* spp. shown by Gram stain can be misleading. Warthin-Starry stain is more useful.

*B. quintana* is known for its fastidious nature, making it difficult to culture from valve tissues. In addition, blood cultures have a sensitivity of <20% for detecting *Bartonella* spp. (*11*). Blood cultures and valve tissue cultures of this patient were both negative. In such cases, clinicians must rely on alternative diagnostic methods, such as serologic tests, molecular techniques, and specialized staining methods, to identify the pathogen. Those methods include the use of PCR, NGS, and Warthin-Starry staining, which can detect *Bartonella* DNA or highlight bacterial clusters that might otherwise be missed (*2*).

Of note, pauci-immune glomerulonephritis has been associated with infectious endocarditis, including *B*. *quintana* (*12*,*13*). The patient’s glomerulonephritis might have been the first manifestation of the *B*. *quintana* infection. This hypothesis is supported by resolution of the glomerulonephritis and no requirement for further immunosuppression after the patient received treatment for the *B*. *quintana* infection.

Globally, *B*. *quintana* endocarditis is on the rise, and the long interval (≈5 months) between symptom onset and manifestation leads to delayed diagnosis (*14*,*15*). As a deadly disease with nearly 10% case-fatality rate, *B*. *quintana* infection requires early diagnosis, before endocarditis occurs, as well as vigilance and clinical suspicion in both at-risk population and patients without known risk factors living in areas with reported cases, such as in this case.

In summary, we report a rare and unexpected case of *B*. *quintana* endocarditis coupled with pauci-immune glomerulonephritis in a patient without known risk factors in Los Angeles, California, USA, highlighting that infection can occur in the general population without a history of homelessness. The diagnosis was challenging and made definitively through extensive testing and investigation. Clinicians should be aware *B*. *quintana* can cause endocarditis and pauci-immune glomerulonephritis in a broader population.
